# A Study of Basal Cell Carcinoma in South Asians for Risk Factor and Clinicopathological Characterization: A Hospital Based Study

**DOI:** 10.1155/2014/173582

**Published:** 2014-11-03

**Authors:** Sumir Kumar, Bharat Bhushan Mahajan, Sandeep Kaur, Ashish Yadav, Navtej Singh, Amarbir Singh

**Affiliations:** ^1^Department of Dermatology, Venereology & Leprology, Guru Gobind Singh Medical College & Hospital, Sadiq Road, Faridkot, Punjab 151203, India; ^2^Department of Skin & V.D., OPD Block, Guru Gobind Singh Medical College & Hospital, Sadiq Road, Faridkot, Punjab 151203, India; ^3^Department of Pathology, Guru Gobind Singh Medical College & Hospital, Sadiq Road, Faridkot, Punjab 151203, India

## Abstract

*Objectives*. Although the incidence of skin cancers in India (part of South Asia) is low, the absolute number of cases may be significant due to large population. The existing literature on BCC in India is scant. So, this study was done focusing on its epidemiology, risk factors, and clinicopathological aspects. *Methods*. A hospital based cross-sectional study was conducted in Punjab, North India, from 2011 to 2013. History, examination and histopathological confirmation were done in all the patients visiting skin department with suspected lesions. *Results*. Out of 36 confirmed cases, 63.9% were females with mean ± SD age being 60.9 ± 14.2 years. Mean duration of disease was 4.7 years. Though there was statistically significant higher sun exposure in males compared to females (*P* value being 0.000), BCC was commoner in females, explainable by intermittent sun exposure (during household work in the open kitchens) in women. Majority of patients (88.9%) had a single lesion. Head and neck region was involved in 97.2% of cases, with nose being the commonest site (50%) with nodular/noduloulcerative morphology in 77.8% of cases. Pigmentation was evident in 22.2% of cases clinically. Nodular variety was the commonest histopathological variant (77.8%). *Conclusions*. This study highlights a paradoxically increasing trend of BCC with female preponderance, preferential involvement of nose, and higher percentage of pigmentation in Indians.

## 1. Introduction

Jacob Arthurin 1827 first coined the term “rodent ulcer” to describe what we now know as a basal cell carcinoma (BCC) [1]. It is the most common cutaneous malignancy worldwide, accounting for 65–75% of all skin cancers. Gross differences are noted in the percentage of skin cancer in the Asians (2–4%) and Blacks (1-2%) as compared to the Caucasians (35–40%) [2]. Although the incidence of skin cancers in India is lower as compared to the Western world, absolute number of cases may be significant due to large population. The existing literature on BCC in India is scant with lack of clinical studies with statistical analysis [3]. So, this study was undertaken to fill this deficit in literature of BCC with focus on epidemiology, risk factors, and clinical and pathological aspects of the disease.

BCC is a nonmelanocytic skin malignancy arising from basal cells of the epidermis or follicular structures and is seen mostly on sun exposed areas, especially head and neck, occasionally over the trunk and limbs, and rarely on the palms, soles, mucous membranes, and genitals [4, 5].

The anatomic distribution of BCC correlates with embryonic fusion planes. Recently, it has been indicated that BCC occurrence is higher along embryonic fusion planes as compared to other areas of the midface, evidence that supports this hypothesis for BCC pathogenesis [6].

Ninety-five percent of these neoplasms occur in patients aged more than 40 years, although cases in childhood and congenital basal cell epitheliomas have been reported [7–9]. In children, it is usually associated with a genetic defect, such as basal cell nevus syndrome, xeroderma pigmentosum, nevus sebaceous, epidermodysplasia verruciformis, Rombo syndrome, or Bazex syndrome.

Sunlight is the most frequent association with development of BCC; risk correlates with the amount and nature of accumulated exposure, especially during childhood. A latency period of 20–50 years is typical between the time of ultraviolet (UV) damage and BCC clinical onset. Both UVB radiation and UVA radiation contribute to the formation of BCC. UVB is believed to play a greater role in the development of BCC than UVA [10]. In a 2012 systematic review and meta-analysis of 12 studies with 9328 cases of nonmelanoma skin cancer, Wehner et al. found that indoor tanning was associated with a significantly increased risk of both basal and squamous cell skin cancers. The risk was highest among users of indoor tanning before age 25 [11]. Apart from UVR, X-ray and Grenz ray exposure is also linked with development of BCC.

Arsenic has been used as a medicinal agent, predominantly the Fowler solution of potassium arsenite, which was used to treat many disorders, including asthma and psoriasis, and is linked to the risk of development of multiple malignancies after a long latency period spanning many years.

The risk of developing new nonmelanoma skin cancer is reported to be 35% at 3 years and 50% at 5 years after an initial skin cancer diagnosis [12]. A study among adults in the United States reports a strong association between excessive alcohol drinking and higher incidence of sunburn, suggesting a linkage between alcohol consumption and skin cancer [13].

## 2. Methods

A hospital based study was conducted at a tertiary care hospital situated in Punjab, North India, from 2011 to 2013. Patients of all ages attending skin outpatient department with suspected lesions were screened for BCC after taking an informed written consent. Patients with histopathologically confirmed BCC were enrolled in the study.

Detailed history with recording of various patient variables like age, gender, duration of symptoms, Fitzpatrick skin phototype, skin color, average daily sun exposure (hours/day), occupation, residence place (rural or urban), exposure to chemicals including pesticides, radiation exposure history, treatment with psoralen UVA (PUVA) or narrow band UVB (NBUVB), smoking, alcohol intake, history of personal or family history of skin cancers, personal or family history of other cancers, history of genetic disorder like xeroderma pigmentosum, albinism, and history of previous treatment.

Clinical examination was done with data collection on various tumor variables which included the following: size, location, number, morphological subtype, and pigmentation. For descriptive purposes, the lesions were classified based on size into small (less than 1 cm in diameter), medium (1-2 cm in diameter), and large (>2 cm in diameter).

Investigations included complete blood count with differentials, bleeding time, clotting time, renal function tests, liver function tests, and viral markers. Additional investigations were done depending upon the clinical scenario. Diagnosis was confirmed by histopathological examination of biopsy specimen with documentation of histopathological variant. To analyze the results, descriptive statistics such as mean, standard deviation (SD), and frequency tables were utilized. Various analytic tests such as *χ* ^2^ test, *P* value, and *t*-test were used. *P* value less than 5% was considered as significant.

## 3. Results

### 3.1. Demographic Data

A total of 36 histopathologically confirmed cases of BCC were enrolled in the study from 2011 to 2013. An increase was seen in absolute number of cases diagnosed per year with 9, 11, and 16 patients in 2011, 2012, and 2013, respectively (Figure 6).

Out of these patients, males were 36.1% (13/36) and females were 63.9% (23/36) with M : F being equal to 0.57 : 1 (Figure 7). Age of the affected cases ranged from 29 to 92 years of age. The mean ± SD age of the patients was 60.9 ± 14.2 years (65.92 ± 14.35 years for males and 57.96 ± 13.54 years in case of females). Although the difference in mean age between males and females was not statistically significant (data was analyzed using unpaired *t*-test), it carries a clinical relevance as females tend to seek medical care earlier than males for suspicious, asymptomatic, and cosmetically disfiguring lesions. The greatest number of patients was in the age group of 61–80 years (47.2%) followed by 41–60 years (38.95%), 21–40 years (8.3%), and 81–100 years (5.6%), respectively (Figure 8). The youngest age of presentation in case of females was 29 years, while in males the corresponding age was 45 years. Correlation between gender and age group was not statistically significant (Fisher exact test *P* value being 0.177), implying that these two variables are independent (Table 1).

Out of all patients, 69.4% (25/36) hailed from rural areas. Majority of the patients were illiterate (80.6%) (Table 2). A statistically significant association was seen between duration of disease and illiteracy (*χ* ^2^ value = 6.95 and *P* value = 0.01). This meant that illiterate patients present at a later stage of disease attributable to lack of awareness about disease entity. Farming was the main occupation among male patients (92.3%), while housekeeping was the major job among female patients (95.7%).

### 3.2. Clinical Data

The duration of disease before seeking medical care ranged from 5 months to as long as 15 years, with mean duration being 4.7 years. The average duration of sun exposure was 6 hours/day in case of males and 2.91 hours/day in female patients. This difference in duration of sun exposure was statistically significant (*t*-value being 6.71 and *P* value = 0.000) (Table 3). However, the females were intermittently exposed to high intensity sunlight due to work in open kitchens and fields during sowing and harvesting seasons.

None of the patients had been taking photoprotective measures such as use of sunscreens and protective clothing. There was no history of treatment with PUVA or NBUVB in any of the study cases. All the patients were nonalcoholics and nonsmokers. No patient had features suggestive of genodermatoses associated with predilection for cutaneous malignancies like xeroderma pigmentosa, albinism, and so forth. Out of 36 patients, one (2.8%) had been previously treated for breast and endometrial carcinoma. Family history of cutaneous and systemic malignancies was not present in any of them. All the cases belonged to Fitzpatrick skin types III and IV (calculated via Fitzpatrick scoring scale).

**Figure 1:**
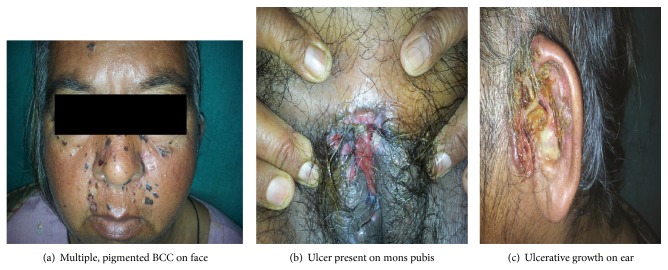
Metastatic BCC.

Majority of patients (88.9%) had a solitary lesion. Out of total 36 patients, two (5.6%) had 2 lesions at the time of presentation. One female (2.8%) patient had multiple lesions over face followed by development of ulcerative growths on ear and mons pubis. She was later diagnosed with BCC having noncontiguous and distant cutaneous metastasis (Figure 1). Another female patient had multifocal lesions on eyelid, temple, and nose. This patient was previously treated for breast and endometrial carcinoma.

**Figure 2:**
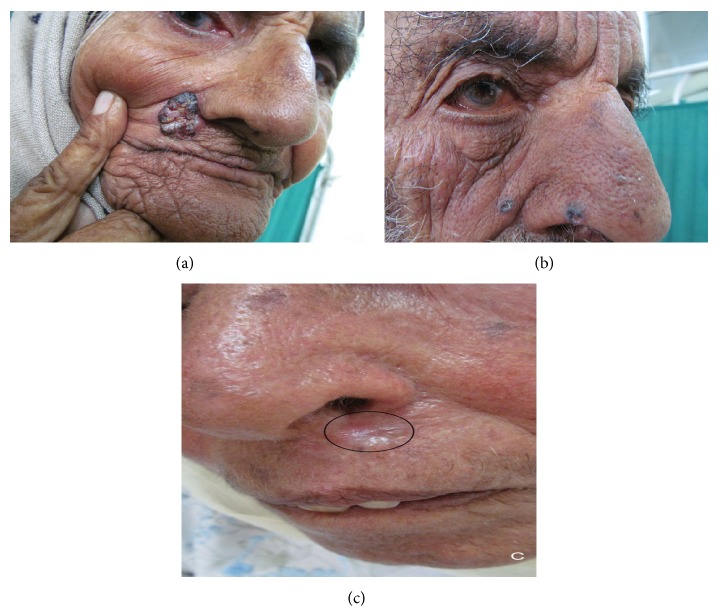
(a) Noduloulcerative, pigmented BCC in an elderly female. (b) Multiple, pigmented papular BCC on face. (c) Morpheaform BCC in an elderly woman.

The size of lesions ranged from as small as 0.5 cm to 5 cm in diameter. The size of lesions was found to have a statistically significant association with duration of disease (*χ* ^2^ = 11.10; *P* value = 0.004) (Table 4). Majority of cases (97.2%) had lesions confined to head and neck area. The distribution of lesions was as follows: nose (50%), cheeks (22.2%), ear and preauricular area (13.9%), lower eyelid (13.9%), temporal area (5.6%), upper lip (2.8%), forehead (2.8%), scalp (2.8%), and mons pubis (2.8%) (Table 5). Nose was the most significant site of involvement in our study (*χ* ^2^ = 14.43; *P* value = 0.01, while corresponding values without involving nose as site of BCC were 1.00 and 0.80). The most common morphological subtype of BCC was nodular/noduloulcerative growth (77.8%) (Figures 1 and 2). A significant percentage of BCC was clinically pigmented (22.2%) (Table 6). Other types observed were micronodular (19.4%) and morpheaform (2.8%) BCC.

### 3.3. Histopathological Data (Figure 3)

The most common histopathological variant was nodular subtype (77.8%) with a significant proportion of tumors being pigmented (16.7%) (Table 7). Other subtypes included basosquamous (8.3%), micronodular (2.8%), morpheaform (2.8%), keratotic (2.8%), and adenoid (2.8%) BCC and BCC with adnexal differentiation (2.8%).

## 4. Discussion

Basal cell carcinoma occurs worldwide (Table 8). So far, BCC has been considered as the disease of the White [14]. Consequently, most of the studies have focused on White populations in Europe, USA, and Australia with scarcity of data from developing countries (Table 9). Estimates of the incidence of BCC are imprecise since there is no cancer registry that collects data on BCC.

Although incidence rates of BCC vary significantly according to the ethnicity and geographic location, most studies show a rising trend in its incidence worldwide. This has been largely attributed to fair complexion and ozone layer depletion resulting in increased UV radiation reaching earth's surface. Similar increasing trend was noticed in our study as well. But factors other than the mentioned above need to be searched and verified as darker skin complexion in Indians should otherwise be protective against BCC. Moreover, ozone layer destruction is most evident over the temperate and polar regions, while India is a tropical country [15, 16].

Basal cell carcinoma is rare in young populations. An increased incidence has also been noticed in children and young adults [17]. This finding highlights the need for early institution of UV protection and skin cancer screening in the pediatric and young adult population. However, there was no case below the age of 20 years in our study. Radiotherapy is another risk factor for the development of BCC in younger age group. Relative risk of BCC is more for children who have undergone radiation therapy for enlarged thymus [18] or neoplasms such as medulloblastoma [19].

**Figure 4:**
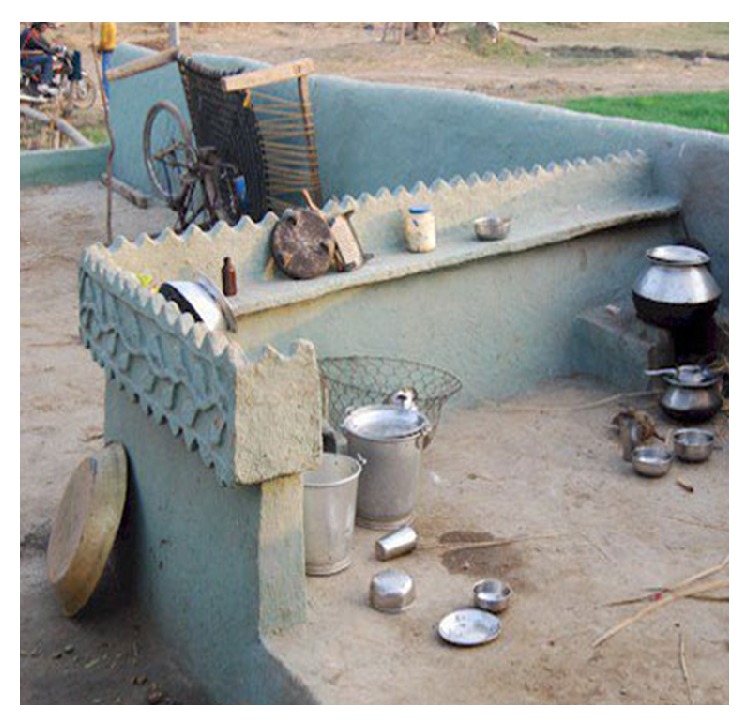
Open kitchen prevalent in rural India.

**Figure 5:**
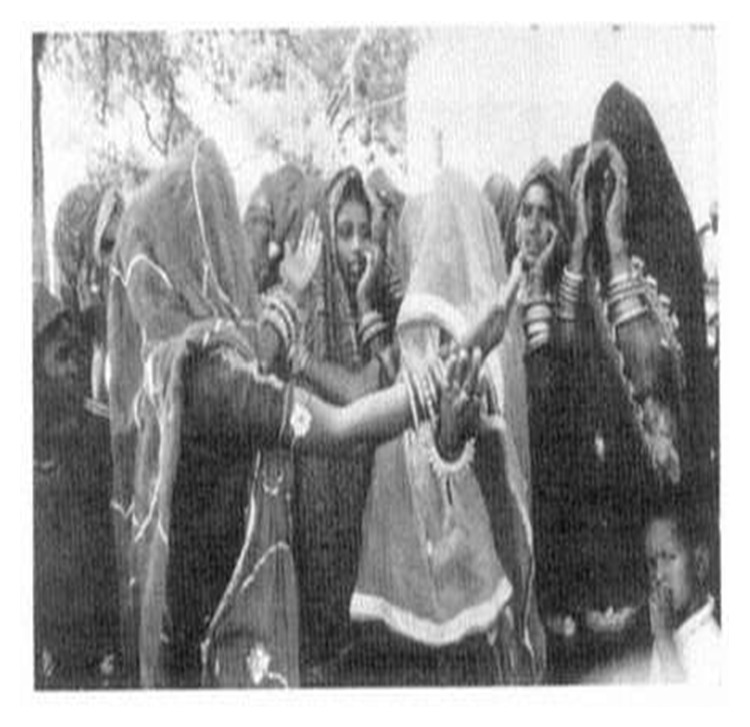
“Veil” custom (obsolete now): an old cultural practice in India in which face is kept hidden by a piece of cloth.

**Figure 6:**
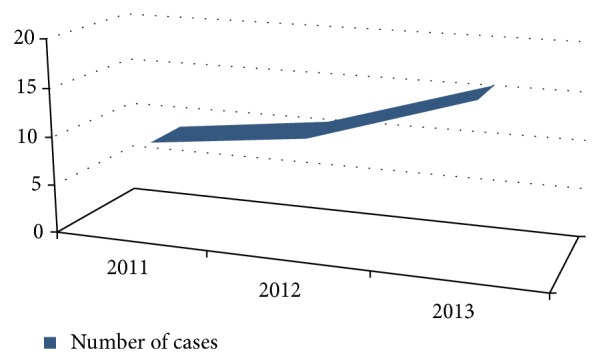
Year-wise diagnosed cases of BCC.

**Figure 7:**
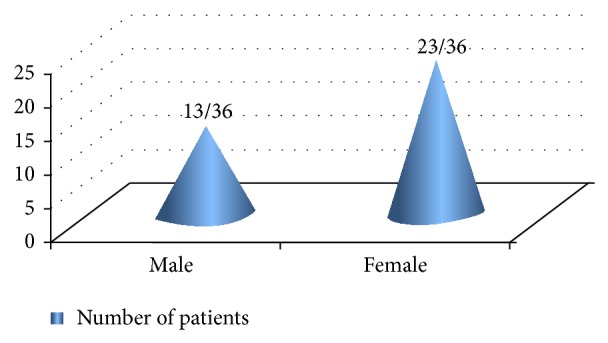
Gender distribution of BCC.

**Figure 8:**
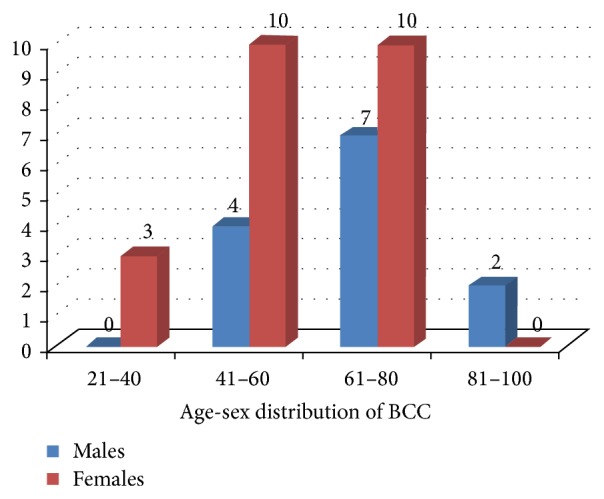
Age-sex distribution of BCC.

BCCs are more common in males as reported in most studies worldwide, presumably due to greater occupational and recreational exposure to ultraviolet radiation (UVR). However, an unusual female preponderance was noticed in our study which is consistent with findings of another Indian series [20]. Indian housewives especially rural women work in open kitchen (Figure 4) during their household chores and work in the fields during sowing and harvesting seasons exposing them to intermittent, high intensity UVR. It might explain higher frequency of BCC in females in our study as intermittent rather than constant, cumulative UVR exposure is implicated in the pathogenesis of BCC [21]. This female predilection may also be attributed to the changes in cultural practices like “veil” custom (Figure 5), structurally thinner skin with lower collagen density in the dermis when compared to men.

The most commonly affected age group in our study was 61–80 years (47.2%) followed by 41–60 years (38.95%), 21–40 years (8.3%), and 81–100 years (5.6%). This closely resembles the findings of another study conducted in East India [22]. The cases belonged to age ranging from 29 to 92 years which is similar to study published from North India [23]. Higher rates of occurrence of BCC among elderly may be due to cumulative UVR induced DNA damage [24] as well as reduced efficiency of immune-surveillance and DNA repair mechanisms with aging [25].

In our study, higher frequency among rural inhabitants was seen when compared to urban residents. This can be explained on the basis of more outdoor activities (as agriculture is the main occupation), changes in clothing preferences, illiteracy, and infrequent use of sunscreens. The rural patients regard initial lesions of BCC as a minor cosmetic problem with insignificant impact on health and seek medical advice only when lesions become symptomatic or disfiguring. So, late presentation to health facilities is equally contributory. A study done in Punjab regarding cancer found that tap water contains high content of arsenic, chromium, iron, and mercury, whereas ground water has abundance of arsenic, chromium, nickel, and iron. Even pesticides have been detected in the locally grown vegetables as well. Tseng et al. found a dose-dependent relation between arsenic levels in drinking water and the prevalence of skin cancers [26]. Thus, exposure to harmful metals and pesticides may also add to the risk of skin cancers, but further clinical and research studies are needed to confirm their role in the pathogenesis of BCC and to delineate underlying mechanisms. Occupations at risk of BCC that are highlighted in our study include agricultural workers and housekeepers.

Use of tanning beds is associated with increased risk of BCC [27]. But their use is uncommon in our part of the world.

Immunocompromised patients like HIV positive patients or organ transplant recipients have a markedly increased risk of both typical and highly invasive NMSC. Interesting fact is that while the BCC/SCC incidence ratio is approximately 4 : 1 in the general population, SCCs become more prevalent in the transplant population with a BCC/SCC ratio of approximately 1 : 2. However, there was no HIV positive case seen amongst our study subjects.

The association between BCC and smoking remains unclear. Some studies refute any relationship between smoking and risk of BCC [28], while Boyd et al. found that BCC in young women is linked with past or current smoking [29]. Smith and Randle described an increased prevalence of BCC larger than 1 cm in diameter among the smokers [30]. However, all the subjects in our study were nonsmokers.

Other iatrogenic risk factors associated with BCC include treatment with psoralens and UVA therapy and NBUVB for various ailments. None of the patients in our study had received such treatments. In a retrospective, nation-wide cohort study of patients with psoriasis subjected to climatotherapy at the Dead Sea, it was found that the overall risk of cancer in patients treated at the Dead Sea was higher than that expected in the general population, owing to an excess risk of nonmelanoma skin cancer (NMSC), with multiple lesions being more common and predilection for younger individuals [31].

The most common location in our study was head and neck area (97.2%) which is close to what was observed by Malhotra et al. [23]. A significant proportion of cases were clinically pigmented (22.2%). This finding was in contrast to what has been seen in Western countries. This pigmentation might be misleading as, clinically, BCC may be misdiagnosed as melanoma.

Although most BCCs are slow-growing, relatively nonaggressive tumors, a minority have an aggressive behavior with local tissue destruction and, rarely, metastasis. Metastatic BCC has a reported incidence of only 0.0028–0.5% [32]. Risk factors for development of metastatic BCC include large primary tumor (>2 cm), location in head and neck region, long standing lesion, multiple primary tumors and recurrences, prior radiation therapy, large tumor depth, invasion of perineural space and blood vessels, fair skin, male gender, and immunosuppression [33]. One of our patients was detected with metastatic BCC (Figure 1).

## 5. Conclusions

This study highlights a paradoxically increasing trend of BCC with female predilection and higher percentage of pigmented lesions in Indians. This skin malignancy tends to be commoner in rural and agriculture based population. Major contributory risk factors include intermittent rather than constant UV exposure, cultural and lifestyle changes, cosmetic indifference, possible role of arsenic and pesticides, improved clinical awareness, and diagnostic facilities. The increasing cancer burden calls for the need of introduction of national screening program including mandatory annual skin examination by trained health professionals at the national level. Since early detection and treatment of lesions are crucial to decrease functional and cosmetic morbidity and costs, this study highlights the importance of improving awareness among general practitioners, public health workers, and general population. The clinical and epidemiological data collected in this study would serve as a reference for future research and may be helpful in the development of preventive and educational strategies.

## Figures and Tables

**Figure 3:**
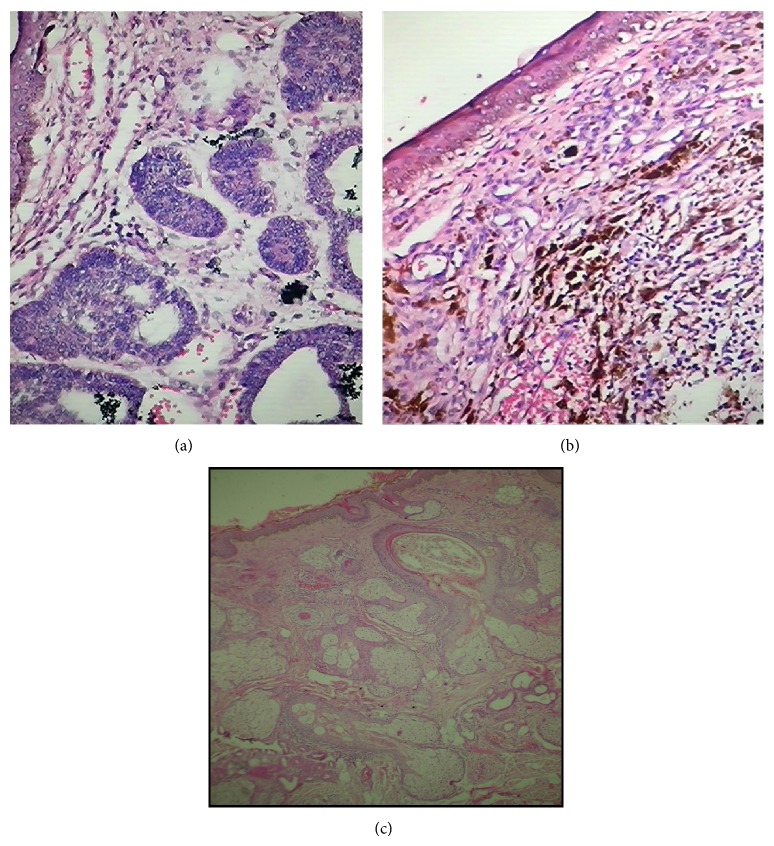
(a) Histopathology (hematoxylin and eosin, 40x): nodular BCC. (b) Histopathology (hematoxylin and eosin, 40x): pigmented BCC. (c) Histopathology (hematoxylin and eosin, 10x): adenoid BCC.

**Table 1:** Age-sex distribution of BCC.

Age (years)	Males	Females	Total	Fisher exact test
				*P* value
21–60	4	13	17	0.177^NS^
61–100	9	10	19	
Total	**13**	**23**	**36**	

For application of appropriate statistical tests, only two age groups were considered.

NS: not significant at 5% level of significance.

**Table 2:** Association between duration of disease and educational status.

Duration of disease (years)	Educational status		Total	*χ* ^2^ value (d.f.)	*P* value
	Educated	Illiterate			
0–5	7	13	20	6.95 (1)	0.01^s^
More than 5	0	16	16		
Total	**7**	**29**	**36**		

S: significant at 5% level of significance.

**Table 3:** Sex-wise comparison of duration of sun exposure.

Gender	Number (*n*)	Mean duration (hours/day) of sun exposure	Standard deviation SD	*χ* ^2^ value (d.f.)	*P* value
Male	13	6	1.15	6.71 (1)	0.000^HS^
Female	23	2.91	1.41		

HS: highly significant.

**Table 4:** Association between duration of disease and size of lesion.

Duration (years)	Small size (<1 cm)	Medium size (1-2 cm)	Large size (>2 cm)	Total	*χ* ^2^ (d.f.)	*P* value
0–5	7	9	4	20	11.10 (2)	0.004^S^
More than 5	0	5	11	16		
Total	**7**	**14**	**15**	**36**		

S: significant at 1% level of significance.

**Table 5:** Distribution of BCC according to site.

Site	*n*	% age of patients	*χ* ^2^ (d.f.)	*P* value
Nose	18	50	14.43 (4)	0.01^s^
Cheeks	8	22.2		
Ear, preauricular area	5	13.9		
Lower eyelid	5	13.9		
Others	6	16.8		
Total	**42**	**100**		

S: significant at 5% level of significance.

**Table 6:** Morphological types of BCC.

Morphology	*n*	% age of patients	*χ* ^2^ value (d.f.)	P value
Nodular/noduloulcerative	28	77.8	37.636 (3)	0.000^HS^
Pigmented	8	22.2		
Micronodular	7	19.4		
Morpheaform	1	2.8		
Total	**44**	**100**		

HS: highly significant.

**Table 7:** Histopathological variants of BCC.

Histopathological variant	*N*	% age of cases	*χ* ^2^ value (d.f.)	*P* value
Nodular	28	77.8	21.14 (2)	0.000^HS^
Pigmented	6	16.7		
Others	8	* *22.3		
Total	**42**			

HS: highly significant.

**Table 8:** Worldwide incidence of BCC.

Country	Incidence of BCC (per 100,000 person-years)	Comment
Australia	>1600	Highest incidence
North America	~300	
Europe	40–80	
Africa	<1	Lowest rates

**Table 9:** Studies regarding BCC in South Asia.

						Highlights of the study		
Study	*n*	Duration and type of study	M	F	M : F	Age	Sites	Subtype
Obaidullah and Aslam, 2008 [34]	100	4 years, prospective	45	55	0.8 : 1	Mean age = 56.3 years	—	24 pigmented nodular, 21 nonpigmented nodular, 30 ulcerative, and6 lesions were of morphoeic type.
Asif et al., 2010 [35]	235	3 years, retrospective	53.2%	46.8%	1.2 : 1	32–90 years	Nose: 28.9% Eye: 24.7% Cheek: 20.4%	—
Laishram et al., 2010 [22]	30	5 years, retrospective	—	—	1 : 2	Median age = 70 years; most common age group is 61–70 years.	83.3% on head and neck, with predilection for face	Nodular subtype was the most frequent.
Malhotra et al., 2011 [23]	34	3 years	—	—	1.6 : 1	28 to 102 years. Majority in age group 40–60 years (44%)	91.2% on head and neck, with commonest site being medial/lateral canthus of eye	Most common histology subtype: nodular (64.7%); pigmented clinically (35.2%)
Chow et al., 2011 [36]	225	10 years, retrospective	94	132	0.7	Mean age = 73.1 (22–100) years	Nose: 31.6% Cheek: 16.5%	Ulcer: 64.8% Nodule: 19.3%
Deo et al. [3]	14	8 years, retrospective	—	—	—	—	—	—
Moore and Bennett, 2012 [37]	10	9 years, retrospective	5	5	—	68.9 years	100% on head region	Nodular: 50% Infiltrative: 10% Sclerosing: 10%
Janjua and Qureshi, 2012 [38]	171	3 years, retrospective	100	71	1.4 : 1	22–90 years (mean 61.3 ± 13.07 years)	Most common site: nose (31.5%) followed by cheek (26.9%)	Nodular variety: 46.2% and pigmented type: 18.7%
Chang and Gao, 2013 [39]	243	8 years, retrospective	118	125	0.94 : 1	65.16 ± 12.62 years	Head and neck region was the most common site (77.4%)	Nodular: 53.9% Superficial: 18.9% Infiltrative-morphoeic: 18.5%
